# SEP Montage Variability Comparison during Intraoperative Neurophysiologic Monitoring

**DOI:** 10.3389/fneur.2016.00105

**Published:** 2016-06-30

**Authors:** Christine Hanson, Athena Maria Lolis, Aleksandar Beric

**Affiliations:** ^1^Department of Neurology, Division of Clinical Neurophysiology, New York University School of Medicine, New York, NY, USA

**Keywords:** IOM, SEP, IOM recording montages, montage comparison, spine surgery, SEP reliability

## Abstract

Intraoperative monitoring is performed to provide real-time assessment of the neural structures that can be at risk during spinal surgery. Somatosensory evoked potentials (SEPs) are the most commonly used modality for intraoperative monitoring. SEP stability can be affected by many factors during the surgery. This study is a prospective review of SEP recordings obtained during intraoperative monitoring of instrumented spinal surgeries that were performed for chronic underlying neurologic and neuromuscular conditions, such as scoliosis, myelopathy, and spinal stenosis. We analyzed multiple montages at the baseline, and then followed their development throughout the procedure. Our intention was to examine the stability of the SEP recordings throughout the surgical procedure on multiple montages of cortical SEP recordings, with the goal of identifying the appropriate combination of the least number of montages that gives the highest yield of monitorable surgeries. Our study shows that it is necessary to have multiple montages for SEP recordings, as it reduces the number of non-monitorable cases, improves IOM reliability, and therefore could reduce false positives warnings to the surgeons. Out of all the typical montages available for use, our study has shown that the recording montage Cz-C4/Cz-C3 (Cz-Cc) is the most reliable and stable throughout the procedure and should be the preferred montage followed throughout the surgery.

## Introduction

The ultimate goal of intraoperative neurophysiological monitoring (IOM) is to reduce the risk of adverse events that can occur during surgeries that put neural structures at risk in an attempt to prevent permanent neurological injury. Somatosensory evoked potentials (SEP) have been used for IOM since their inception in the late 60s and 70s (1–5). Through the years, the field of IOM has been improved by the addition of other modalities, such as electromyography (EMG) and motor evoked potentials (MEP); however, SEP recording have continued to remain the gold standard. False-positive SEP changes during surgery can transiently occur due to various causes, such as change in anesthesia delivery, blood loss, blood pressure, body temperature, accumulation of anesthetic in the patient’s body, and various technical interferences in the operating room. For the purposes of conducting IOM, it is very important to distinguish real SEP changes from sources of interference, such as technical problems or noise, which may lead to an increase in number of false positive changes.

Somatosensory evoked potentials are usually recorded with multichannel derivations of cortical and subcortical responses. Cz′, C3′, and C4′ are situated according to the modified 10–20 system for EEG electrode placement: Cz′ is located 2 cm posterior to Cz, C3′ (left) and C4′ (right) 7 cm from Cz′ on a line connecting it with the external auditory meatus. For the purposes of this study, Cz′ will be referred to as Cz. C3′ and C4′ will be referred to as Cc, if contralateral to stimulated extremity and Ci when ipsilateral to stimulated extremity in this article. The locations of these traditional recording electrodes Cc (upper extremity) and Cz (lower extremity) represent the location of the arm and leg area primary somatosensory cortex within the postcentral gyrus. Cz can be used bilaterally for recording of tibial and peroneal SEPs since the leg areas are located in depth of the interhemispheric fissure on the postcentral gyrus very close to one another. The C3′Fz/C4′Fz is the montage that is typically used for obtaining the cortical responses of the upper extremity. Since the first description of peroneal nerve SEPs and tibial nerve SEPs, Cz′Fz is the montage used for SEP recording of lower extremity responses (6, 7).

Beric and Prevec in 1981 discovered a peculiar tibial nerve cortical generator behavior and in 1983 published that the tibial nerve SEP exhibits asymmetrical scalp amplitude distribution (8, 9). The positively charged sensory leg generator is situated slightly lateral to Cz in the hemisphere contralateral to the stimulated leg. Isopotential lines around it appear to be distorted toward the hemisphere ipsilateral to the stimulated leg. Therefore, Ci is partially active (positive) while Cc appears to represent a negatively charged area. The question whether this is due to the presence of at least two generators or to the existence of a dipole (9–16) has yet to be resolved. The responses of tibial and peroneal nerves are historically recorded in analogy to the location of the recording electrodes for median and ulnar SEPs. Fz is used as reference and Cz, representing the leg area on the somatosensory cortex, as recording electrode. Interestingly, the distribution of potentials evoked by stimulation of the tibial and peroneal nerves suggests CiCc and CzCc as good possible montage alternatives to CzFz. This can be achieved using the preexisting electrodes already placed for routine upper and lower extremity SEP monitoring. Cz is positively charged, Ci partially active (positive), Cc negatively charged, and Fz practically inactive. Hence, in theory, the amplitude of P40 for CiCc should be in the same range as in the CzFz derivation. For the CzCc montage, the difference between the two electrodes becomes even more positive over a differential amplifier; hence, P40 amplitude would here be expected to be the largest.

Most default IOM machine protocols and most surgical centers seem to be using CzFz or CiCc montages for cortical IOM traces. Existing guidelines by the American Clinical Neurophysiology Society and by the American Society of Neurophysiological Monitoring suggest that the use of multichannel recording should be performed while conducting IOM in case of technical problems. According to the guidelines, different derivations, such as CiCc and CzCc, should be considered in order to determine and select the trace with the highest amplitude (17–19). However, the selection of these montages at baseline does not take into account the development of the recordings throughout the surgery. The goal of this study was to find the most appropriate combination of the least number of montages that gives the highest yield of monitorable surgeries. We wanted to analyze and compare these three montages, CzFz, CiCc, and CzCc in regard to their appearance at baseline as well as their development throughout the surgery. Amplitude size is important for SEP IOM, but the consistency during the surgery leading to stable and reliable monitoring avoiding major fluctuations in amplitude and latency is even more important. This especially plays a role during the monitoring if the routinely used montage has lower amplitudes at baseline or shows poorly repeatable baseline recordings. Our study takes this into account, since we believe that possible improvement of SEP reliability during the surgery by using different electrode montages can be of benefit for the patient.

## Materials and Methods

This prospective study evaluated 524 consecutive patients undergoing surgery at NYU Langone Medical Center from January 1, 2010 through December 31, 2010 who did not exhibit any postoperative neurologic deficits. Patient’s ages ranged from 8 to 84. All patients had undergone an instrumented spinal fusion surgery with neurophysiologic intraoperative monitoring. Surgical procedures included chronic neurologic and neuromuscular conditions, such as congenital or degenerative spine disease, scoliosis, myelopathy, and spinal stenosis, that were indicated to undergo lumbar spinal fusion, cervical spinal fusion, and scoliosis correction surgery. In all cases, the tibial nerve SEP was followed during IOM, and when indicated, peroneal nerve SEP was also performed. The tibial nerve is the largest distal lower extremity nerve with the greatest representation on the cortex and is used for monitoring of the posterior spinal columns of the spinal cord. The tibial nerve was used for lower extremity SEP monitoring for spinal surgery involving cervical and thoracic levels as well as for monitoring of all surgeries with lumbar spinal instrumentation. For surgery involving level L5 and lower, peroneal nerves were monitored additionally. Three cortical montages CzFz, CiCc, and CzCc were set up for lower extremity SEP recording. This is the standard recordings montage and protocol that is routinely performed for instrumented spinal fusions at our institution. At first, 150 patients underwent detailed analysis. After those results were reviewed, another 374 patients were added, using a quicker approach, based on the initial analysis of the first 150 patients. A total of 524 patients with 1048 tibial traces and 546 peroneal traces were assessed.

### Stimulation and Recordings

For stimulation of all nerves, silver-chloride disposable stick-on electrodes were utilized. To stimulate the tibial nerve, the stimulating electrodes were placed behind the medial malleolus. The peroneal nerve was stimulated at the anterolateral aspect of the ankle. For stimulation of both nerves, the cathode was always placed proximally. Nicolet Endeavor CR (Natus, Middleton, WI, USA) was used in all cases of neurophysiologic monitoring. The nerves were stimulated using rectangular pulses of 500 μs duration and stimulation frequency was 3.1 Hz. The time base was 100 ms. Stimulation intensity was adjusted to get the largest amplitude tracings using the smallest necessary intensity, with the maximal intensity of 35 mA.

The recordings were obtained by using sterile stainless steel subdermal EEG electrodes placed in the scalp. Needles were positioned at Fz, Cz′, C3′, C4′ according to the modified international 10–20 system for EEG electrode placement. Cz′, therefore, was placed above the leg cortical representation area (1.5–2 cm behind Cz). C3′ and C4′ were located above the arm areas (7 cm from Cz′ on a line connecting it with the external auditory meatus) of the postcentral somatosensory cortex. The resistances were kept below 5 kΩ. Traces were recorded with a 20–1000 Hz filter and amplified 10,000 times. SEP activity was displayed by the Endeavor software on a Windows PC. Electrode montages set up for recording were CzFz, CiCc (Ci being the electrode C3′ or C4′ ipsilateral to the stimulated leg nerve), and CzCc (Cc was C3′ or C4′ above contralateral hemisphere). One hundred responses were averaged. Depending on the stage of the surgery, stimulation was applied every 5–20 min.

### Detailed Approach of SEP Analysis

The first groups of patients were analyzed based on a detailed approach. The first 150 patients, which were 300 tibial and 156 peroneal SEPs recordings, were analyzed in regard to 5 categories: clarity, repeatability, size (amplitude) of response, consistency over the time course of the operation, and level of noise during the procedure. They were given a point for each category, adding up to a score between 5. Clarity, repeatability and size of response were measured at baseline. Consistency and noise (absence of noise) were taken into consideration as they developed throughout the IOM, including the closing trace. Trace in this study is operationally defined as individual montage recording. Criteria for the trace scorings are shown in Table 1. Montages were compared indirectly according to the five categories and their quality for IOM was determined according to their scores (indirect approach).

**Table 1:** **Criteria for waveform classification of SEP traces in five categories based on a 1-5 scale**.

	1	2	3	4	5
Clarity	Absent waveform	Present waveform, but not clear P40 and/or N50	Present waveform, clear P40 and N50	Clearly present entire waveform	Perfect W waveform
Repeatability	Not repeatable	Poorly repeatable	Repeatable P40 and N50	Repeatable entire waveform	Identical waveform
Amplitude	<0.2 μV	0.2–0.4 μV	0.4–0.8 μV	0.8–2.0 μV	>2 μV
Consistency	Not present after surgical opening	Present most of the time – needs repeated averaging	Always present, but with variable latency and/or amplitude – less than alarm criteria	Similar waveform throughout, same latency and similar amplitude	Almost identical waveform throughout IOM
Noise	Noise throughout IOM	Repeated noise interfering with waveform recognition	Repeated noise not interfering with waveform recognition	Occasional noise not interfering with waveform recognition	No noise

**Figure 1:**
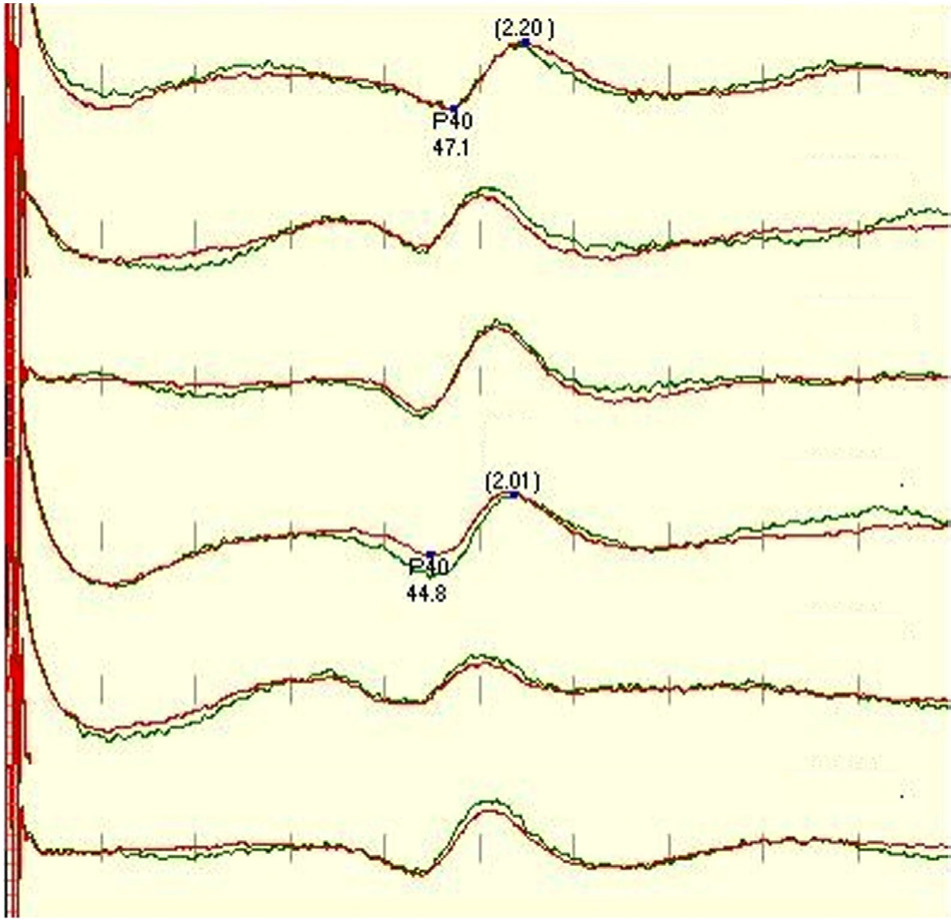
**Standard protocol for tibial SEP, recorded (in order) from CzFz, CiCc, and CzCc montage (first 3 traces left leg-tibial nerve stimulation, traces 4–6 right leg – tibial nerve stimulation)**. All montages show comparably good traces. Marked are P40 latency and peak-to-peak amplitude between P40 and N50. P40 latencies of CiCc and CzCc vary from CzFz. CzCc presents with nicer traces, but all three montages would serve well for IOM. Green trace is the baseline, and the red trace is the closing trace of surgery.

We considered recordings satisfactory if their scores were 3 and above and present only if their scores were 2 and above. If the trace was well formed, repeatable, with an appropriate amplitude, consistency, and no noise, it was scored 5 in all categories as the example of the traces in Figure 1. As scoring is subjective and the 1–5 scale is not linear and can, therefore, be potentially misleading, the electrode montages were also compared directly (one on one), using the same five categories but given a point only to the better trace in the corresponding category, the other trace was given 0 points (direct approach). This detailed approach was performed in order to assess which relevant component of a single montage is important for quality of IOM and differs from other used montages.

### Quick Approach for SEP Analysis

After the first data collection of SEP recordings was assessed, the remaining 374 patients SEP recordings (748 tibial nerves and 390 peroneal nerves) were analyzed using a quicker approach. This approach involved comparing traces from three different montages directly at Baseline and according to their Development during the case. At the baseline, the clarity, repeatability, and size were assessed as one result. Consistency and noise were evaluated as they developed throughout the case as the second result. This was followed by taking the SEP recordings of the first group of 150 patients, which was analyzed in the detailed approach, and re-assessing the recordings based on the quicker approach described above. At this point, the data of all 524 patients were statistically analyzed as divided into the following pairs for direct comparison: CzFz vs. CiCc, CzFz vs. CzCc, and CiCc vs. CzCc.

### Statistical Analysis

All data were statistically analyzed using Chi-square tests for direct comparisons of pairs and ANOVA One way tests for comparisons of three groups in indirect montage comparison (detailed approach). We set the significance level α = 1% in order to achieve more reliable results, thus meaning that only results *p* < 0.01 were considered statistically significant. Pearson correlation coefficient was calculated using data from Quick approach only by comparison of baseline traces score vs. development score during the monitoring for each of three montages.

## Results

### Indirect and Direct Comparisons of SEPs in Five Categories (Detailed Approach)

In the first paradigm, 300 tibial nerve recordings and 156 peroneal nerve recordings were analyzed. Each nerve recording used the following montages: CzFz, CiCc, and CzCc. Each montage was given points in the five categories: clarity, repeatability, size of response, consistency during the monitoring, and noise level throughout the monitoring and added up to a score of 1–5. Both separate indirect ANOVA comparisons were made in all five categories, as well as indirect comparison when all five category scores were averaged.

#### Indirect Tibial Nerve SEP Montage Comparison

For tibial monitoring, CzFz montage showed an average score of 2.82 out of 5 points. Both other montages reached better scores than CzFz, with an average of 3.76 for CiCc and 3.79 for CzCc out of 5 points, as shown in Table 2.

**Table 2:** **Usability of traces for IOM**.

		Median score out of 5 points	Traces non-recordable or not satisfying (≤2 on 0–5 scale) (%)	Traces good for IOM (≥3 on 0–5 scale) (%)
Tibial (*n* = 300)	CzFz	2.82	42.0	58.0
	CiCc	3.76	22.7	77.3
	CzCc	3.79	21.0	79
Peroneal (*n* = 146)	CzFz	1.64	79.2	20.8
	CiCc	2.27	64.0	36.0
	CzCc	2.66	53.0	47.0

**Figure 2:**
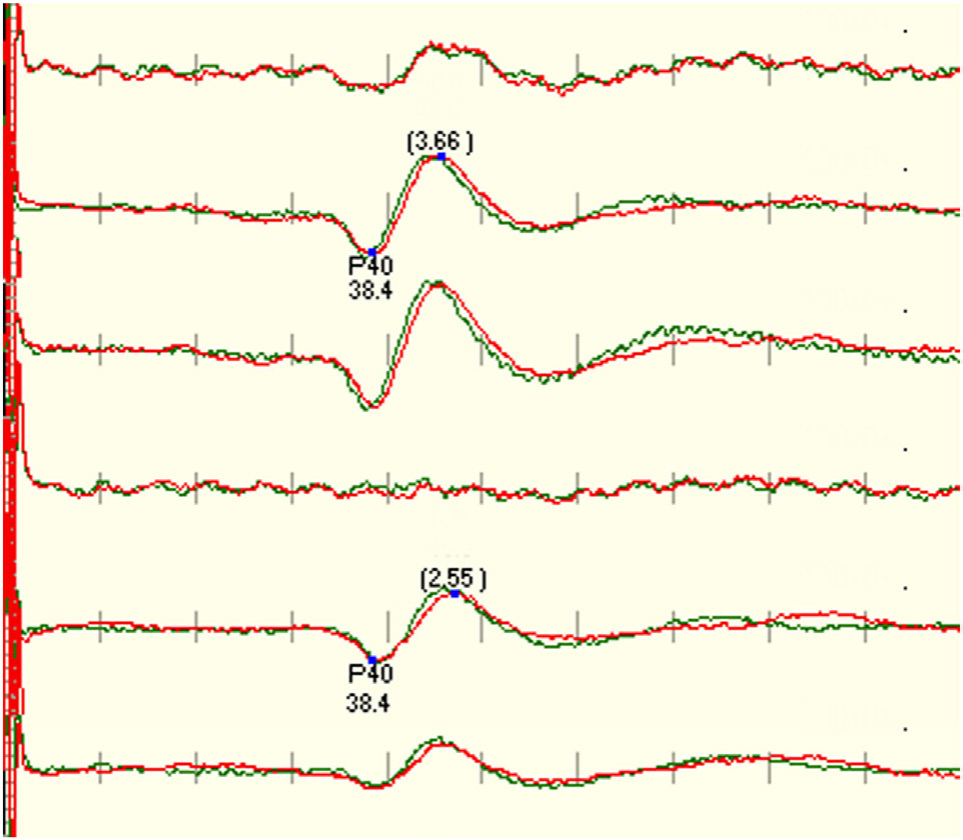
**Example that shows the right tibial SEP is not recordable using CzFz montage**. Due to good recordings in CiCc and CzCc, the monitorability of the surgery was improved. CzFz picked up a lot of noise, while the transverse montages are mainly free from noise.

There was no statistical difference between CiCc and CzCc montage (*p* = 0.610), but both montages were significantly better than CzFz. While there were some response present in 88.7% of CzFz recordings, 96.3% of CiCc recordings, and 96.7% of CzCc recordings (scores above 2), only 58.0% of cases had presented with a CzFz montage that satisfied the intraoperative monitoring (scores ≥3 on 0–5 scale), as shown in Table 2. CiCc montage showed 77.3% and CzCc recordings showed 79.0% with scores above 3, therefore, demonstrating well-monitorable traces in ~20% more cases than CzFz. For tibial SEPs, CiCc and CzCc both presented with traces significantly better than CzFz in all five categories. Figure 2 depicts a high level of noise affecting the CzFz montage, which prevented this from being monitorable, while the traces from other two montages were practically noise free.

**Figure 3:**
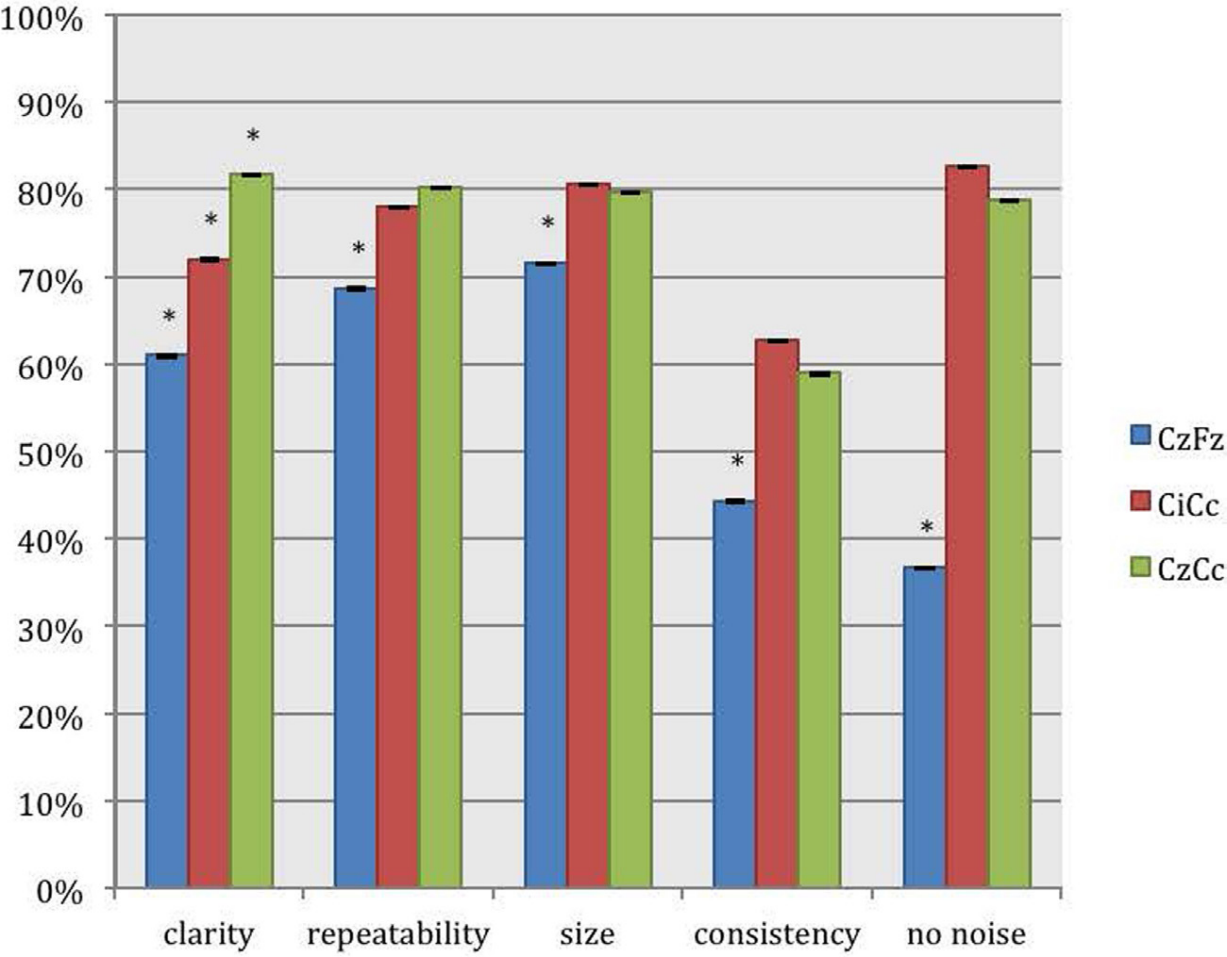
**Direct comparison of the montages CzFz, CiCc, and CzCc for tibial nerve SEPs according to their score in the five categories: clarity, repeatability, size, consistency, and absence of noise**. Asterisks (*) marks montages significantly different from the other montages. All significance levels (Chi-square tests) were *p* < 0.001 with one exception for size of the trace: CzFz < CiCc (*p* < 0.001) and CzFz < CzCc (*p* = 0.003).

The montages CiCc and CzCc showed significant difference only in clarity, as depicted in Figure 3. The differences in the other categories were insignificant. The additional quantitative analysis of size of amplitudes showed no significant difference between the three montages CzFz (mean 0.91 μV), CiCc (mean 0.91 μV), and CzCc (mean 1.02 μV). However, CzCc was the largest.

#### Direct Tibial Nerve SEP Montages Comparison

Tibial SEP direct pair comparisons of the montages CzFz, CiCc, and CzCc confirm the findings of the indirect observations described above in all five categories as shown in Table 3.

**Table 3:** **Direct comparison of the montages CzFz, CiCc, and CzCc in five categories**.

**Tibial (*n* = 300)**		**CzFz (%)**	**CiCc (%)**	**Both equal (%)**
	Clarity	27.7 ± 0.2	32.6 ± 0.1	39.7
	Repeatability	17.3 ± 0.2	**37.0 ± 0.1**	45.7
	Size	18.3 ± 0.2	**44.3 ± 0.2**	37.3
	Consistency	14.7 ± 0.2	**41.3 ± 0.2**	44.0
	Noise	3.7 ± 0.2	**62.3 ± 0.1**	34.0
		**CzFz (%)**	**CzCc (%)**	**Both equal (%)**
	Clarity	13.7 ± 0.2	**51.3 ± 0.1**	35.0
	Repeatability	9.7 ± 0.2	**38.7 ± 0.1**	51.7
	Size	26.3 ± 0.2	**43 ± 0.2**	30.7
	Consistency	11.3 ± 0.2	**34.7 ± 0.2**	54.0
	Noise	4.0 ± 0.2	**53.0 ± 0.1**	43.0
		**CiCc (%)**	**CzCc (%)**	**Both equal (%)**
	Clarity	11.0 ± 0.2	**24.0 ± 0.1**	65.0
	Repeatability	11.3 ± 0.2	**17.7 ± 0.1**	71.0
	Size	25.0 ± 0.2	30.0 ± 0.2	45.0
	Consistency	18.0 ± 0.2	13.7 ± 0.2	68.3
	Noise	**12.0 ± 0.1**	7.0 ± 0.1	81.0
**Peroneal (*n* = 156)**		**CzFz (%)**	**CiCc (%)**	**Both equal (%)**
	Clarity	25.0 ± 0.3	**41.7 ± 0.3**	33.3
	Repeatability	18.6 ± 0.3	**28.8 ± 0.3**	52.6
	Size	28.8 ± 0.3	35.9 ± 0.3	35.3
	Consistency	16.7 ± 0.3	**31.4 ± 0.3**	51.9
	Noise	1.9 ± 0.3	**44.8 ± 0.2**	53.2
		**CzFz (%)**	**CzCc**	**Both equal**
	Clarity	14.1 ± 0.3	**48.1 ± 0.3**	37.8
	Repeatability	11.5 ± 0.3	**39.7 ± 0.3**	48.7
	Size	17.9 ± 0.3	**39.1 ± 0.3**	42.9
	Consistency	9.6 ± 0.3	**37.1 ± 0.3**	53.2
	Noise	3.8 ± 0.3	**39.1 ± 0.3**	57.1
		**CiCc (%)**	**CzCc (%)**	**Both equal (%)**
	Clarity	10.3 ± 0.3	**34.0 ± 0.3**	55.8
	Repeatability	5.8 ± 0.3	**30.1 ± 0.3**	64.1
	Size	16.0 ± 0.3	**30.1 ± 0.3**	53.8
	Consistency	7.7 ± 0.3	**33.3 ± 0.3**	58.9
	Noise	**12.2 ± 0.2**	2.6 ± 0.3	85.3

*Percentages represent which montage was better, unless they are equal. In bold are all statistically significant comparisons*.

CiCc montage was better than CzFz in all categories. These results were significant for repeatability, size, consistency, and noise. CiCc montage showed better results than CzFz; 19.7% better for repeatability, 26% for size, and 26.6% more often better for consistency. It also surpassed CzFz montage by picking up less noise in 58.6% more of the IOMs. CzCc proved to be better than CzFz also in all five categories, and in addition significantly superior for all five categories: CzCc traces presented with clear baselines of 37.6% more often than CzFz were better in size 16.7% more frequent than CzFz, more consistent and repeated better in approximately a third more of the IOMs. Noise was picked up by CzFz 49% more often than by CzCc. As for a comparison between CiCc and CzCc in tibial recordings, the two montages showed only little differences. CzCc was the significantly better montage in 13% more often than CiCc for clarity and 6.4% more often for repeatability. However, CiCc picked up significantly less noise in 5% more of the IOMs than CzCc.

#### Indirect Peroneal Nerve SEP Montages Comparison

The results for peroneal monitoring are similar, and generally all traces had poorer results due to the peroneal nerve being a smaller nerve. CiCc (2.27) and CzCc (2.66) montages of the peroneal nerve reached higher scores than the CzFz (1.64) montage, as shown in Table 2. This result of CzFz being the weakest montage and CzCc showing the best traces out of the three montages was statistically significant.

**Figure 4:**
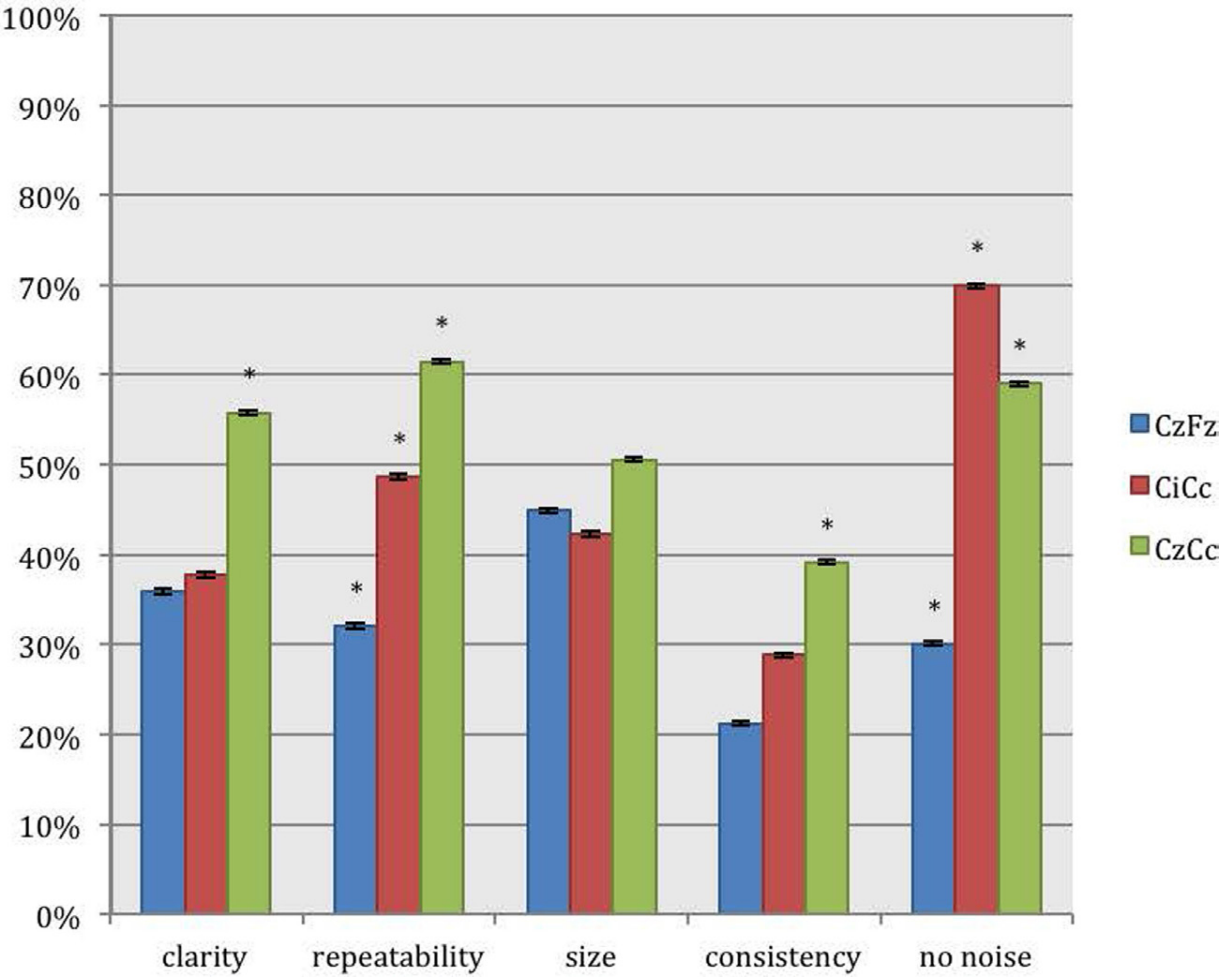
**Direct comparison of the montages CzFz, CiCc, and CzCc for peroneal nerve SEPs according to their score in the five categories: clarity, repeatability, size, consistency, and absence of noise**. Asterisks (*) mark montages statistically significantly different from the other montages. All significance levels (Chi-square tests) *p* < 0.001 with two exceptions: (a) consistency: CzCc vs. CzFz (*p* < 0.001) and CzCc vs. CiCc (*p* = 0.004); (b) absence of noise: CzFz vs. CiCc and CzFz vs. CzCc (*p* < 0.001); CiCc vs. CzCc (*p* = 0.003).

For recordings of the peroneal nerve, CzCc presented with better traces than CzFz in all 5 categories, as shown in Figure 4. This result is statistically significant for all categories but size. According to Figure 4, although CzCc was the significantly better trace according to clarity, repeatability, size (*p* = 0.036), and consistency (*p* = 0.004), CiCc picked up significantly less noise than CzCc (*p* = 0.003). While differences between CzFz and CiCc were significant only for repeatability and noise, CiCc also showed a tendency to be the better montage for consistency. The additional quantitative analysis of size of amplitudes showed that there was no significant difference between the three montages CzFz (mean 0.61 μV), CiCc (mean 0.53 μV), and CzCc (mean 0.53 μV).

#### Direct Peroneal Nerve SEP Montages Comparison

Direct comparison of the montages showed similar results for intraoperative peroneal nerve SEPs. CiCc and CzCc showed clear advantage over CzFz in all five categories: CiCc presented with better traces than CzFz in 16.7% more of the IOMs for clarity, was 10.2% more repeatable, showed larger traces in 7.1% more of the IOMs, was more consistent in 14.7% more of the IOMs and presented with less noise in even 42.9% more of the IOMs, as shown in the lower part of Table 3.

CzCc recorded better traces than CzFz in even 34% more considering clarity, was more repeatable 28.2%, showed larger amplitudes 21.2% more often, stayed more consistent throughout the operation in 27.5% more of the IOMs and presented with less noise 35.3% more often.

When compared with the tibial nerve recordings where differences between CiCc and CzCc were relatively minor, peroneal nerve SEP montage CzCc had a very clear advantage over the CiCc montage. CzCc traces showed better clarity, larger amplitude, better repeatability, and consistency, 23.7, 14.1, 24.3, and 26.3%, respectively, more frequent than CiCc recordings. However, only the noise level was significantly higher in CzCc traces and CiCc picked up less noise in 9.6% more of the IOMs. All differences (with the exception of size difference) between CzFz and CiCc were significant.

### Direct Comparison of Baseline Recordings and Their Development during Monitoring (Quick Approach)

After this detailed analysis of the first 150 patients, another 748 tibial SEP recordings and 390 peroneal SEPs recordings of the remaining 374 patients were assessed and their electrode montages CzFz, CiCc, and CzCc were ranked at baseline as well as according to their development throughout the surgery. Same approach was used on the first 150 patients and for general comparison of the usability and reliability of these three montages for IOM, all 524 patients were evaluated.

#### Tibial Nerve SEP Montages Comparison

This direct comparison showed for the 1048 tibial nerve SEPs recordings that CiCc presented with better traces twice as often as CzFz. CzCc recorded better traces 4 times as often as CzFz. Comparing the CiCc and CzCc montages, CzCc showed better recordings in 11.1% more of the IOMs. All of these results: CzFz vs. CiCc, CzFz vs. CzCc, and CiCc vs. CzCc were significant, as shown in Table 4.

**Table 4:** **Overall pair wise comparison of three montages CzFz, CiCc and CzCc in all 524 patients**.

		CzFz vs. CiCc			CzFz vs. CzCc			CiCc vs. CzCc		
		CzFz	CiCc	Equal	CzFz	CzCc	Equal	CiCc	CzCc	Equal
Tibial (*n* = 1048)	Better trace (%)	26.7	56.3	17.0	15.8	63.1	21.1	26.4	37.5	36.1
	Chi-square test	*p* < 0.001*			*p* < 0.001*			*p* < 0.001*		
Peroneal (*n* = 546)	Better trace (%)	27.3	48.0	24.7	20.7	51.5	27.8	19.4	45.6	35.0
	Chi-square test	*p* < 0.001*			*p* < 0.001*			*p* < 0.001*		

CzFz was not monitorable in 142 (13.5%) of all tibial SEP IOMs, while CiCc was non-monitorable in only 82 IOMs (7.8%) and CzCc could not be recorded in 80 IOMs (7.6%). Thus, almost half of the cases with non-recordable CzFz traces became monitorable by using CiCc and CzCc montages. Out of the 142 IOMs where no trace could be recorded over CzFz, 79 traces (55.6%) turned out to be monitorable by using CiCc montage and 75 traces (52.8%) by using CzCc montage. There also existed some cases in which CzFz was monitorable but CiCc (32 traces, 3.1% of all IOMs) or CzCc (29 traces, 2.8% of all IOMs) could not be followed. In 0.9% of the tibial nerve recordings, as an extreme, CzFz showed a good quality trace at baseline (score 4 or 5), but turned non-monitorable (score 1) during the procedure, while CiCc and/or CzCc stayed monitorable and could be followed. So, overall, combining all three montages, 94.3% of all IOMs were monitorable.

#### Peroneal Nerve SEP Montages Comparison

Results for the 546 peroneal nerves showed the same trend: CiCc montage recorded almost double as often better traces than CzFz montage, CzCc was in over 30% more of the IOMs better than CzFz. Comparing CiCc and CzCc montages, CzCc presented with the better trace in 26.2% more IOMs than CiCc.

Peroneal SEPs were not monitorable in a much higher number of IOMs than tibial nerve recordings in every of the three montages. In over a third of all IOMs (37%), CzFz was not monitorable. CiCc could not record traces in 26.2% and CzCc presented as non-recordable in 23.6% of the IOMs. This shows a number of IOMs in which CzFz montage was non-monitorable, the peroneal nerve recording continued to be monitorable by adding CiCc and CzCc montages. CiCc was able to provide monitoring for additional 83 (40.8%) and CzCc for additional 78 (38.4%) out of the 203 IOMs where no trace was recordable with CzFz. However, in some cases CiCc (42 traces) and CzCc (34 traces) were non-monitorable although CzFz could be used for IOM. As an extreme, in 2.6% of the peroneal recordings, CzFz recorded a good quality trace at baseline (score over 3) but then deteriorated to a point where it was no longer monitorable while at least one of the other montages stayed monitorable.

### Correlation of Baseline Recordings and Development Throughout the Monitoring

**Figure 5:**
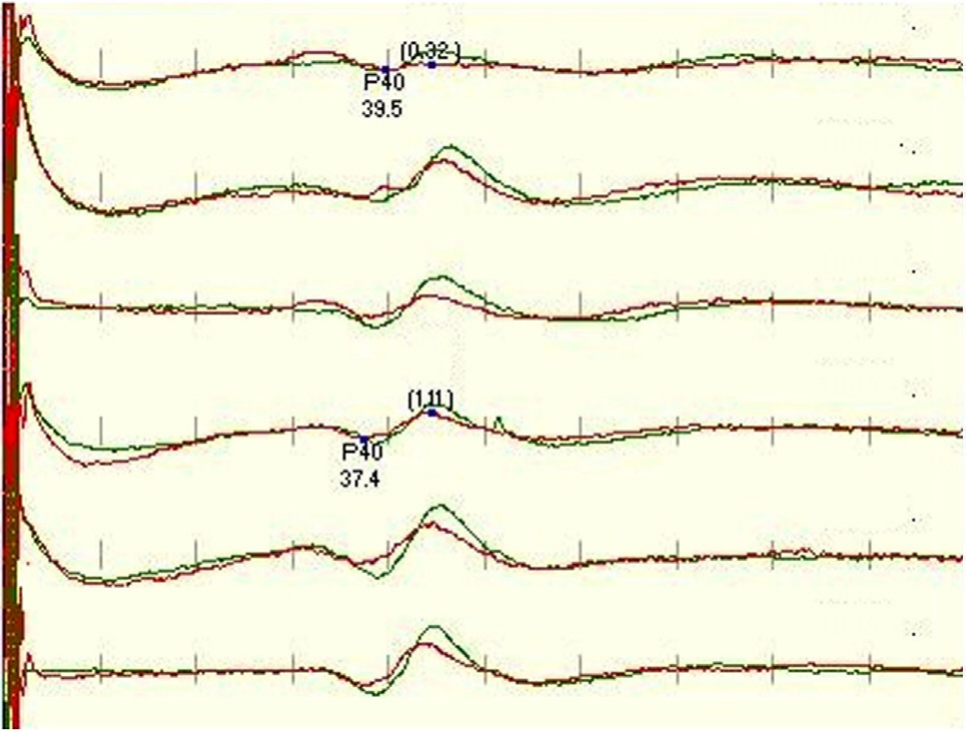
**Figure depicts a significant deterioration of the left leg-tibial nerve SEP trace recorded with CzFz montage (trace 1) with red being on-going recording compared with the green baseline recording**. An acute reduction of >50% in amplitude is a sign for possible neurological damage and typically needs to be reported to the surgeon. As the right tibial SEP CzFz (trace 4) recording stayed stable, this unilateral change implicates even more strongly that it is a true and reportable SEP change. However, this example shows that both transverse montages are minimally affected, especially CiCc (trace 2) which is rather stable. This stability in the other two montages ultimately prevents the neurophysiologist from interrupting the surgery and alarming the surgeon.

Our study also looked at predicting whether an IOM recording would have well-formed SEPs throughout the monitoring, based on the quality of the baseline recordings. When the ranking of the traces at baseline was compared with the development throughout the cases, we found only moderate correlation according to Pearson’s correlation coefficient for tibial (rCzFz = 0.59, rCiCc = 0.62, rCzCc = 0.60) and peroneal (rCzFz = 0.58, rCiCc = 0.49, rCzCc = 0.46) nerve SEPs. This means that it cannot be predicted at baseline which montage will be best monitorable throughout the surgery, supported with an example in Figure 5.

## Discussion

Despite the addition of other monitoring modalities, SEPs continue to be the standard modality used in IOM for various reasons, most importantly that they can be run frequently, and are not susceptible to overstimulation and neuromuscular blockade use. An improvement in their reliability and/or increase of the monitorable cases could be of great benefit for IOM.

The nerve most frequently recorded for SEP IOM is the tibial nerve, as it is the largest sensory nerve of the leg with the greatest area of representation in the somatosensory cortex. Therefore, it is generally used to assess the post central gyrus for brain surgery, the posterior column of the spinal cord in operations where the spinal cord is involved, and to assess the nerve roots in lumbar cases. This is generally achieved by monitoring SEPs using the CzFz electrode montage. Due to the paradoxical distribution of leg SEPs, it is recommended in recent guidelines to consider CiCc and CzCc as additional electrode montages for intraoperative monitoring. According to our data, all montages are equally acceptable in around 17–36% of the IOMs. However, in the remainder of the IOMs where the patient had poor recordings with small amplitudes and/or delayed latency, more reliable monitoring is needed in order to detect true changes. Our study shows that CzCc and CiCc montages seem to be more appropriate than CzFz, as shown in Tables 2 and 4.

Peroneal traces often have very small amplitudes so that the recordings become unstable during the time course of the operation. This is due to the peroneal nerve being a small nerve at the level of the ankle where we stimulate it. For lumbar spine surgery with instrumentation of the levels L5 and lower, we are especially concerned with foot drop and the peroneal nerve SEP should additionally be recorded. Monitoring peroneal nerves in addition to tibial nerves serves as redundancy check and provides higher reliability for IOM. If traces change simultaneously in both nerves, there is a very strong implication for a true positive event along the ascending pathway. Changes in only one nerve while the other one stays stable can be very specific, but makes the result more questionable. For the reasons named above, increasing the reliability of intraoperative peroneal SEP monitoring as well as reducing the number of non-monitorable cases would be of great value for IOM.

### CiCc and CzCc as Additional Derivations for IOM

CzFz is historically used as the standard montage for intraoperative monitoring of leg SEPs. However, according to Table 2, which is an overall comparison of montages and their usability for reliable monitoring, this montage was actually found to provide quality recordings only in 58% of the tibial nerve SEPs and about 20% of the peroneal nerve SEPs. When the overall comparison of all montages against themselves was performed as depicted in Table 4, it corroborated our earlier findings that there are other montages more superior to CzFz for IOM. For tibial nerves, CiCc traces were equally as good as or better than CzFz traces in 73.3% of the IOMs, CzCc showed equally as good or better traces than CzFz in 84.1% of the IOMs. For peroneal SEP, CiCc was equally as good as or better than CzFz in 72.7, CzCc was better or equally as good as CzFz in 79.3.1% of the IOMs. This shows that the monitorability of leg SEPs, particularly that of the peroneal nerve, can definitely be improved by additional montages, such as CiCc, and especially by adding CzCc electrode montage.

This success rate for leg tibial SEP monitoring of 86.5% for Cz-Fz montage is lower than the one described earlier by our group (20) where 93.4% of the leg SEPs recorded with CzFz montage showed a positive result. The population of patients evaluated in that study was predominantly brain surgery patients with no or little preoperative deficit. The patient population in this study consisted mainly of patients suffering from lumbar stenosis, cervical stenosis, radiculopathies, and scoliosis. All of the abovementioned conditions, if severe enough, can easily render SEPs non-recordable. Additionally, there is a possibility of polyneuropathy and foot swelling, which further complicates the monitoring, and can ultimately add to the non-monitorability of cases. Many patients had preoperative deficits and poorly formed traces to start off with at baseline. These cases are prone to show intraoperative fluctuations and it is crucial to be able to distinguish real changes from false positives. Improvement of the SEP recordings with additional electrode derivations is, therefore, of great benefit for IOM and ultimately of benefit for the patient. With addition of the two transverse montages (CiCc and CzCc), the success rate of tibial nerve SEP monitoring increased to 94.3%, which is even slightly better than that in our previous study.

### Amplitude, Noise, and Consistency of SEP Monitoring

We were not able to find a statistical difference between the sizes of amplitudes when comparing the three montages. These results somewhat vary from those of MacDonald et al. (21) who found their optimal derivation to be significantly larger than CzFz and, therefore, improve the signal-to-noise ratio (22). This may be due to the different patient population, as well as that we used standard electrode placements instead of lengthier search for maximum amplitudes. We evaluated many patients with preoperative neurologic deficit who presented with poor SEP traces. However, size evaluation alone is not enough to determine the optimal derivation. As long as the amplitudes are greater (0.4 μV), clarity of waveform, noise, and especially consistency throughout the monitoring are even more important for IOM.

In the environment of the operating room, different sources of interference can affect the monitoring traces and make them unreliable or non-monitorable. Noise can occur at any point in time during the surgery, affecting different channels or montages differently, not only at the baseline. Usually, there is at least one montage that may pick up less noise than others. In our study, CzFz traces seem to be most prone to pick up these interferences during the surgery. Although CiCc and CzCc both showed less noise interference than CzFz in around 50–60% (tibial nerve) and 40–45% (peroneal nerve) of the cases, their traces can be affected as well. In our study, CzCc montage picked up noise only 5% more often than CiCc traces. Generally, SEP recordings are more likely to be affected by noise and interferences with larger distance between their recording and reference electrodes. This is a possible explanation for why CzFz and even more so when more frontal references such as PFz are used, tibial and peroneal nerve recordings appear noisier than CiCc and CzCc in so many cases. With Cz and Cc being slightly less apart than Ci and Cc electrodes, it remains unexplained why CiCc is the montage with the slightly better results according to noise level. It may, however, have to do with the generator orientation between recording and reference electrodes on one side and localization of the sensory leg area on the cortex in depth of the interhemispheric fissure. Changes in waveform, amplitude, and latency may also occur during the surgery. This is often due to fluctuations in anesthesia, temperature, or blood pressure. These inconsistencies surprisingly do not always occur over all montages. Therefore, one or more traces might decrease in amplitude or show delays in latency. It is difficult to predict, which montage will be the one that will change according to the baseline amplitude. Therefore, the largest amplitude montage does not necessarily predict the best consistency during the entire surgical procedure. As long as at least one other trace is not affected, changes in one montage do not need to be reported to the surgeon. It appears that CzCc is the least affected by all these changes. There is no good explanation for such often temporarily occurring changes. Possible causes include interference with the OR surrounding and anesthetic effects that lead to “wandering topography” of the optimal recording site. MacDonald suggested this to be caused by changes in the scalp topography due to intraoperative brain shifts of the cerebral volume and location (23). This suggestion does not explain the changes seen in our population, as they did not contain any craniotomies.

### The Importance of Multiple Montages, Especially CzCc for SEP Recordings in IOM

Adding other montages for monitoring can decrease the number of false positives. Tibial CiCc and CzCc were around 15–20% more reliable than CzFz recordings. For peroneal SEPs, CiCc showed 7.6% more consistent traces than CzFz and CzCc showed even better consistency than CiCc in another 17.9% of cases. Furthermore, we were able to lower the number of non-monitorable cases by ~50% for tibial traces and 20% for peroneal traces. This was achieved by adding CiCc and CzCc montages to the standard CzFz montage. CiCc seems to pick up the least amount of noise, while CzCc is usually the sturdiest trace. Although we were able to show that CiCc and CzCc traces are superior to the ones recorded by CzFz in many cases, the best electrode montage for a particular surgery can vary individually. There is only moderately significant correlation between baseline and development throughout the IOM. This can sometimes make it very difficult to predict at baseline which of the traces will stay most consistent or pick up the least amount of noise. Therefore, it is important to have more electrode montages for cortical recording from baseline on, and follow them all. Adding more channels and montages can help improve monitoring by filling gaps of inconsistency in other traces.

It is very common at many centers to use only one montage of electrodes for intraoperative cortical SEP recording. Oftentimes, subcortical montages are added for higher intraoperative reliability since these traces are generally less affected by anesthetics, blood pressure, and changes in temperature. Subcortical montage can be used for monitoring of spinal surgeries such as we routinely record, while they are not useful for monitoring of cranial cases as they are below the level of surgery and could be used only for monitoring of proper stimulation input. There is no question that multiple channel recordings provide more information and, therefore, higher reliability for intraoperative SEP monitoring. Although cervical traces are usually well defined with reasonably large amplitudes for arm SEP recording, they are often not very clear for leg recordings. In many cases, this leaves the monitoring technician with one effective montage CzFz only, for IOM. Any change in this montage, therefore, results in having to alarm the surgeon. Adding more cortical montages gives more traces to be compared and more reliability throughout the case and can ultimately lead to calling less false-positive changes.

As we were able to show, adding transverse montages (CiCc, CzCc) improves IOM reliability, reduces the number of non-monitorable cases, and therefore is expected to reduce calling for false positives. CiCc and CzCc proved to be more consistent throughout the procedures. This is especially important for monitoring patients who suffer from severe spinal stenosis and myelopathies. A spectrum of abnormalities during surgeries can be observed; from slight decrease to over more than 50% deterioration of CzFz montage traces and even to turning to non-recordable. As already discussed non-monitorable cases could be reduced by over 50% for tibial and around 20% for peroneal recording. Non-monitorable cases have a higher risk for complications than monitorable cases (24). Therefore, any and every increase in the number of monitorable cases is important. Our study showed how especially important this was for peroneal SEPs as its monitorability was improved from 53 to 74%. Still there a small number of cases in which CzFz montage trace is recordable but CiCc and CzCc traces show poor or no response. These cases would be missed by choosing transverse derivations only for cortical SEP recording and omitting CzFz. Therefore, we suggest a combination of transverse and longitudinal montages for IOM, covering CzFz, CiCc, and CzCc if the number of channels available allows doing so.

If there is a restriction in channels, CzCc and CzFz montages should be recorded as CzCc proved to be the overall best trace of the three and CzFz as standard montage has proven its value for IOM over decades. Yet, if only one channel is available, CzFz should be replaced by CzCc. CzCc is still better in most qualities than CiCc. A small body of evidence is starting to build naming CzCc a better single channel choice than CzFz (21–25). It might be sensible to omit the subcortical trace, when not satisfying or monitorable, and create space for an additional cortical montage recording. Ultimately, if more channels are available, multichannel recording of all three montages CzFz, CiCc, and CzCc with addition of cervical recording could and should be used throughout the case. More cases would become reliably monitorable and the number of non-monitorable cases would be reduced. This is of benefit for surgery and in patients’ best interest.

As a result of the findings of the data analysis that has been presented in this report, our approach to performing intraoperative monitoring has changed. We now routinely follow Cz-Cc as the primary montage throughout the surgery, and use the other two montages as backup. This is a departure from our prior practice of using Cz-Fz as the primary montage.

## Author Contributions

CH, AL, and AB all made substantial contributions to the conception and design of this original research. CH performed the analysis of the data, whereas CH, AB, and AL performed the interpretation of the data. The work was drafted by CH, and revised by AB, CH, and AL who are in agreement to be accountable with the final version to be published.

## Conflict of Interest Statement

The authors declare that the research was conducted in the absence of any commercial or financial relationships that could be construed as a potential conflict of interest.

## References

[1] Larson SJ Sances S Jr Evoked potentials in man. Neurosurgical applications. Am J Surg (1966) 111(6):857–61. 5326593 10.1016/0002-9610(66)90189-9

[2] Nash CL Brodkey JS Croft TJ A model for electronical monitoring of spinal cord function in scoliosis patients undergoing correction (abstract). J Bone Joint Surg Am (1972) 54:197–8.

[3] Nash CL Lorig RA Schatzinger LA Brown RH Spinal cord monitoring during operative treatment of the spine. Clin Orthop Relat Res (1977) 12:100–5. 598095

[4] Macon JB Polettu CE Conducted somatosensory evoked potentials during spinal surgery. Part 1: control conduction velocity measurements. J Neurosurg (1982) 57(3):349–53. 10.3171/jns.1982.57.3.0354 7097330

[5] Nuwer MR Dawson E. Intraoperative evoked potential monitoring of the spinal cord: enhanced stability of cortical recordings. Electroencephalogr Clin Neurophysiol (1984) 59:318–24. 10.1016/0168-5597(84)90049-2 6203721

[6] Cracco RQ Spinal evoked response: peripheral nerve stimulation in man. Electroencephalogr Clin Neurophysiol (1973) 35(4):379–86. 10.1016/0013-4694(73)90195-8 4126811

[7] Jones SJ Small DG Spinal and sub-cortical evoked potentials following stimulation of the posterior tibial nerve in man. Electroencephalogr Clin Neurophysiol (1978) 44(3):299–306. 10.1016/0013-4694(78)90305-X 76536

[8] Beric A Prevec TS. The early negative potential evoked by stimulation of the tibial nerve in man. J Neurol Sci (1981) 50(2):299–306. 10.1016/0022-510X(81)90175-1 7229671

[9] Beric A Prevec TS. Distribution of scalp somatosensory potentials evoked by stimulation of the tibial nerve in man. J Neurol Sci (1983) 59(2):205–14. 10.1016/0022-510X(83)90038-2 6854350

[10] Rossini PM Cracco RQ Cracco JB House WJ. Short latency somatosensory evoked potentials to peroneal nerve stimulation: scalp topography and the effect of different frequency filters. Electroencephalogr Clin Neurophysiol (1981) 52:540–52. 10.1016/0013-4694(81)91429-2 6172254

[11] Vas GA Cracco JB Cracco RQ. Scalp-recorded short latency cortical and subcortical somatosensory evoked potentials to peroneal nerve stimulation. Electroencephalogr Clin Neurophysiol (1981) 52:1–8. 10.1016/0013-4694(81)90182-6 6166447

[12] Cruse R Klem G Lesser RP Lueders H Paradoxical lateralization of cortical potentials evoked by stimulation of the posterior tibial nerve. Arch Neurol (1982) 39:222–35. 10.1001/archneur.1982.00510160028005 7073532

[13] Yamada T Machida M Kimura J Farfield somatosensory evoked potentials after stimulation of the tibial nerve. Neurology (1982) 32:1151–8. 10.1212/WNL.32.10.1151 6889700

[14] Emerson RG Pedley TA Somatosensory evoked potentials. 2nd ed In: Daly DD Pedley TA, editors. Current Practice of Clinical Electroencephalography. New York: Raven Press (1990). p. 679–706.

[15] Yamada T. Neuroanatomic substrates of lower extremity somatosensory evoked potentials. J Clin Neurophysiol (2000) 17(3):269–79. 10.1097/00004691-200005000-00005 10928639

[16] Terada K Umeoka S Baba K Sakura Y Usui N Matasuda K Generators of tibial nerve somatosensory evoked potential: recorded from the mesial surface of the human brain using subdural electrodes. J Clin Neurophysiol (2009) 26(1):13–6. 10.1097/WNP.0b013e3181969043 19151613

[17] Nuwer MR Daube J Fischer C Schramm J Yingling CD Neuromonitoring during surgery. Report of an IFCN committee. Electroencephalogr Clin Neurophysiol (1993) 87(5):263–73. 10.1016/0013-4694(93)90179-Y 7693437

[18] Toleikis JR American Society of Neurophysiological Monitoring. Intraoperative monitoring using somatosensory evoked potentials. A position statement by the American Society of Neurophysiological Monitoring. J Clin Monit Comput (2005) 19(3):241–58. 10.1007/s10877-005-4397-0 16244848

[19] American Clinical Neurophysiology Society. Guideline 9A: guidelines on evoked potentials. J Clin Neurophysiol (2006) 23(2):125–37. 10.1097/00004691-200604000-00010 16612230

[20] Chen X Sterio D Ming X Para DD Butusova M Tong T Success rate of motor evoked potentials for intraoperative neurophysiological effects of age, lesion location, and preoperative neurologic deficits. J Clin Neurophysiol (2007) 24(3):281–5. 10.1097/WNP.0b013e31802ed2d4 17545833

[21] MacDonald DB Stigsby Z Al Zayed Z A comparison between derivation optimization and Cz’-FPz for posterior tibial P37 somatosensory evoked potential intraoperative monitoring. Clin Neurophys (2004) 115:1925–30. 10.1016/j.clinph.2004.03.008 15261872

[22] MacDonald DB Al Zayed Z Stigsby B. Tibial somatosensory evoked potential intraoperative monitoring: recommendations based on signal to noise ratio analysis of popliteal fossa, optimized P37, standard P37, and P31 potentials. Clin Neurophys (2005) 116:1858–69. 10.1016/j.clinph.2005.04.018 16005261

[23] MacDonald DB. Individually optimizing posterior tibial somatosensory evoked potential P37 scalp derivations for intraoperative monitoring. J Clin Neurophysiol (2001) 18(4):364–71. 10.1097/00004691-200107000-00008 11673702

[24] Thuet Padberg AM Raynor BL Bridwell KH Riew KD Taylor BA Increased risk of postoperative neurologic deficit for spinal surgery patients with unobtainable intraoperative evoked potential data. Spine (2005) 30(18):2094–103. 10.1097/01.brs.0000178845.61747.6a 16166902

[25] Miura T Sonoo M Shimizu T. Establishment of standard values for the latency, interval and amplitude parameters of tibial nerve somatosensory evoked potentials (SEPs). Clin Neurophys (2003) 114:1367–78. 10.1016/S1388-2457(03)00094-4 12842736

